# Awareness and Opinions of Research Professionals on India's New Drug and Clinical Trials Regulations: Protocol for a Cross-Sectional Web-Based Survey Study

**DOI:** 10.2196/14744

**Published:** 2020-01-21

**Authors:** Vishal Vennu, Saurabh Dahiya

**Affiliations:** 1 School of Pharmacy Lingaya’s Vidyapeeth Faridabad India; 2 Department of Rehabilitation Sciences College of Applied Medical Sciences King Saud University Riyadh Saudi Arabia

**Keywords:** online survey, professionals, clinical trial, rules

## Abstract

**Background:**

Although several studies have been conducted and several articles have been published on India's new clinical trial regulations, very few have examined the views of investigators and ethics board members regarding modifications to the previous regulations. Overall, they have neglected to find out the opinions of other relevant professionals, such as research assistants, coordinators, associates, and managers. To our knowledge, no study has yet investigated the awareness and opinions of Indian research professionals on the new 2019 regulations.

**Objective:**

This study aims to describe the awareness and opinions of Indian research professionals on the new drug and clinical trial regulations.

**Methods:**

In this cross-sectional, Web-based study, we will conduct an open survey for various Indian research professionals. These professionals will be selected randomly using multiple sources. The survey questionnaires, which have already been validated, were developed using the form function in Google docs. A Web link was generated for participants to take the survey. Descriptive statistics will be shown as means and standard deviations for constant variables, whereas certain variables will instead be shown as numbers and percentages.

**Results:**

The survey was opened in July 2019. Enrollment has already started and will be completed in three months. The results calculations are expected to begin in October 2019.

**Conclusions:**

The results of the survey are expected to represent the views of research professionals on the new regulations that will support the development of clinical research and the pharmaceutical industry in India. These regulations are expected to help advance clinical trials, help with the approval of new drugs, and enhance ethical norms in the country.

**International Registered Report Identifier (IRRID):**

PRR1-10.2196/14744

## Introduction

### Background

In India, the clinical trials industry has exemplified rapid growth in the last few years, driven by economic globalization, and is thus one of the most promising economic sectors in the country [1]. This rapid growth of clinical trials may be attributed to the outsourcing of clinical trials to India by various multinational pharmaceutical organizations [2]. Furthermore, the availability of infrastructure, such as comprehensive treatment, a broad spectrum of prevalent disorders, ethnic variation, English-speaking health care specialists, and medical and information technology, provides proper conditions for conducting clinical trials in the country [2]. However, reports of irregularities in the administration of clinical trials has overshadowed the flourishing of the industry [2,3]. These ethical violations in the industry have exposed loopholes in the regulatory system, which has led to it struggling to oversee clinical trials effectively [4,5].

Due to this, the loopholes of the regulatory system were amended in 2016 by Indian authorities to strengthen the regulatory mechanisms for reviewing clinical trials. These amendments were issued based on a high level of scrutiny, significant media attention [6], the involvement of non-governmental organizations [3], hearings in the Supreme Court, and the recommendations of an expert committee [7]. Details of these three back-to-back amendments and their associated challenges have been summarized elsewhere [8-11]. Furthermore, two pilot studies have detailed the knowledge of investigators and members of ethics boards regarding the impact of these regulations [12,13].

Recently, new regulations were issued in 2019 [14], which involved amending the 2016 regulations [8] to bring further changes in the clinical research sector. These rules have focused on clarifying the terms of clinical trials of new drugs and phase IV trials, and of clarifying post-trial access to new drugs, clinical trial approval validity, equality, compensation, and monitoring, which have all been described elsewhere [15]. To our knowledge, no study has thus far described research professionals’ awareness and opinions regarding these new rules and regulations.

### Objectives

The purpose of this survey is to describe the awareness and opinions of research professionals on the new drug and clinical trials regulations.

### Hypothesis

We hypothesize that most Indian research professionals will be aware of, will welcome new changes to, and will agree that these new changes will speed up clinical trials, new drug approval, and improve ethical standards for clinical trials in the country. Also, they will aid with the expansion of clinical research and the pharmaceutical industry in India.

## Methods

### Overview

The methodology used in this survey is based on previous studies [16-18], and on the Checklist for Reporting Results of Internet E-Surveys (CHERRIES) [19].

### Ethics

The Institutional ethics committee was notified about the intended survey and excused from the review. This study does not require the informed consent of participants because this is an observational survey involving no risk or minimal risk to participants [20].

### Design

A cross-sectional, online survey design will be adopted to collect the data. No data which directly identifies any of the participants will be collected, but data that indirectly identifies participants, such as demographic information (eg, age, gender), will be collected. Mean, or range will be used to represent age, and counts/percentages will be used to show gender. We will select participants from different sources, including databases (eg, Clinical Trial Registry India, Indian Society Clinical Research, and the Central Drugs Standard Control Organization), personal networks, hospitals, institutions, and LinkedIn.

### Participant Eligibility Criteria

Those who are eligible to take part in this survey include research professionals. For this study, a research professional is defined as research investigators, members of ethics committees, assistants, coordinators, associates, and managers who have engaged in clinical trials in India. The number of target participants for this survey will be 80-100. Participants will be chosen randomly to avoid any selection bias.

### Data Collection

Validated questionnaires were selected from previous studies [12,13] and obtained from the respective authors or journals with their approval for use in the present project. Each questionnaire was developed using the form function in Google docs, which is a widely used and free survey tool [21]. A link was generated to enable participants to take the survey online [22]. The Google form feature of a limit of one respondent per email (respondents will be required to sign into Google) was enabled to prevent multiple entries from the same people. The “required to answer” feature was also enabled for each question, except for the following question: “If you are a member of the ethics committee, what is your role?”

The survey was announced on LinkedIn [23]. An email or message with a website link was sent to participants on LinkedIn to request that they complete the online, self-administered questionnaire. Participants' participation in this survey is entirely voluntary and anonymous. Therefore, it does not require participants to provide a name or any other identifying information, except for age and gender. Email reminders will be sent to all the participants to ensure the maximum possible number of responses. Respondents will be able to review and change their answers before clicking on the submit button at the end of the form. All the responses will be accessible by only the study investigator.

### Outcome

The outcome of this will be an enhanced understanding of research professionals’ awareness and opinions of the new clinical trials rules that came into effect in 2019.

### Statistical Analysis

Descriptive statistics will be estimated for continuous variables, whereas counts and percentages will be shown for categorical parameters. These data will be utilized to describe the awareness and opinions of the participants. *Post hoc* tests, such as the independent one-tailed *t* test and chi-square test, will be employed to test the significance of continuous and dichotomized parameters, respectively. Missing data will be explored in future studies, as appropriate. Results will be presented through graphs or tables. An analysis will be carried out utilizing SAS version 9.2 for Windows (SAS Corporation Inc, North Carolina, USA).

## Results

The enrollment of Indian research professionals will begin from multiple sources in July 2019 and is expected to end in November 2019. The results evaluations are expected to start in October 2019. The survey questionnaires being used in this project were previously validated by other studies [12,24], and approval was obtained for their reuse. The demographic characters of the respondents, such as age, gender, and region in India, will be presented in a table. The awareness and opinions of the respondents will be summarized in tables and illustrated as a pie chart.

## Discussion

### Primary Findings

This study, using a Web-based, online, open survey, aims to investigate the awareness and opinions of Indian research professionals who have engaged in clinical trials regarding the new rules put into place in 2019. It is crucial to understand the awareness and opinions of Indian research professionals, as they act as communicators and are a bridge between sponsors and patients. Therefore, understanding the awareness levels and opinions of these research professionals is crucial to determine whether clinical trials are being conducted per the new rules. Furthermore, considering the new directives, these professionals play a crucial role in addressing and reporting unethical clinical trials on vulnerable populations in India.

This open survey will be conducted online, so it will not be necessary to meet participants at any point physically. The survey will need about 10 minutes to complete. Participants will be able to take the survey on their mobile devices or their computers. Participation in this survey will be voluntary and anonymous, which is expected to boost the response rate.

The findings will expand the current knowledge base regarding the awareness and opinions of various Indian research professionals on the new clinical trial regulations, and allow for comparison to the findings of previous studies. Some professionals may have a higher level of awareness of the regulation now or may even have changed their perspectives regarding the recent amendments to Schedule Y of the Drug and Cosmetic Act.

### Limitations

Nonetheless, this survey has some limitations. First, the survey will be taken by only a specific sample within the population. Therefore, the results cannot be generalizable to the whole population of India. Second, self-administered questionnaires may introduce some level of social desirability bias. Finally, the surveys are limited by their cross-sectional design, which allows us to determine the association but not causality.

### Conclusion

At present, there is a significant lack of clarity on the awareness levels and opinions of various clinical research professionals in India regarding the new rules. Findings from this study will fill a glaring gap in the literature by providing a knowledge base regarding views of the new rules. The findings may improve the ethical and quality standards of clinical trials, which will further benefit the expansion of the clinical research and pharmaceutical industry in India by speeding up clinical trials, the approval of new drugs, and protecting patients, especially vulnerable ones.

## Abbreviations

CHERRIES: Checklist for Reporting Results of Internet E-Surveys

## References

[1] Gupta YK, Padhy BM (2011). India's growing participation in global clinical trials. Trends Pharmacol Sci.

[2] Maiti R (2007). Clinical trials in India. Pharmacol Res.

[3] Gulhati CM (2004). Needed: closer scrutiny of clinical trials. Indian J Med Ethics.

[4] Yee A (2012). Regulation failing to keep up with India's trials boom. Lancet.

[5] Gogtay NJ, Ravi R, Thatte UM (2017). Regulatory requirements for clinical trials in India: What academicians need to know. Indian J Anaesth.

[6] Ramamurthy NV (2012). Inept media trials of clinical trials. Perspect Clin Res.

[7] Ministry of Health and Family Welfare (2013). India Environment Portal.

[8] Karwa M, Arora S, Agrawal S (2013). Recent Changes to Clinical Trial Regulation in India: Focus on Serious Adverse Events, Compensation and Registration of Ethics Committees. Pharm Med.

[9] Lahiry S, Sinha R, Choudhury S, Mukherjee A, Chatterjee S (2018). Paradigm shift in clinical trial regulations in India. Indian J Rheumatol.

[10] Saxena P, Saxena R (2014). Clinical trials: changing regulations in India. Indian J Community Med.

[11] Rake B, Haeussler C (2019). Did relaxing clinical trial regulation enhance the stock of scientific knowledge in India? Not necessarily. PLoS One.

[12] Davis S, Sule P, Bughediwala M, Pandya V, Sinha S (2017). Ethics committees and the changed clinical research environment in India in 2016: A perspective!. Perspect Clin Res.

[13] Kadam R, Borde S, Madas S, Nagarkar A, Salvi S, Limaye S (2016). Opinions and perceptions regarding the impact of new regulatory guidelines: A survey in Indian Clinical Trial Investigators. Perspect Clin Res.

[14] Ministry of Health and Family Welfare (2019). The Central Drugs Standard Control Organisation.

[15] Tanya Kapur (2019). Turacoz Healthcare Solutions.

[16] Eysenbach G, Wyatt J (2002). Using the Internet for surveys and health research. J Med Internet Res.

[17] Leece P, Bhandari M, Sprague S, Swiontkowski MF, Schemitsch EH, Tornetta P, Devereaux PJ, Guyatt GH (2004). Internet versus mailed questionnaires: a randomized comparison (2). J Med Internet Res.

[18] Whitehead L (2011). Methodological issues in Internet-mediated research: a randomized comparison of internet versus mailed questionnaires. J Med Internet Res.

[19] Eysenbach G (2004). Improving the quality of Web surveys: the Checklist for Reporting Results of Internet E-Surveys (CHERRIES). J Med Internet Res.

[20] Nijhawan LP, Janodia MD, Muddukrishna BS, Bhat KM, Bairy KL, Udupa N, Musmade PB (2013). Informed consent: Issues and challenges. J Adv Pharm Technol Res.

[21] Travis L (2010). One of Many Free Survey Tools: Google Docs. Journal of Electronic Resources in Medical Libraries.

[22] (2019). Google Docs.

[23] (2019). LinkedIn.

[24] Cagnazzo C, Campora S, Ferretti E, Arizio F, Marchesi E (2017). New European Clinical Trial Regulation: perception and expectations in Italy. Ann Oncol.

